# Ensilability of Biomass From Effloresced Flower Strips as Co-substrate in Bioenergy Production

**DOI:** 10.3389/fbioe.2020.00014

**Published:** 2020-01-31

**Authors:** Jürgen Müller, Juliane Hahn

**Affiliations:** ^1^Group Grassland and Forage Science, Faculty of Agriculture and Environmental Sciences, University of Rostock, Rostock, Germany; ^2^Group Crop Health, Faculty of Agriculture and Environmental Sciences, University of Rostock, Rostock, Germany

**Keywords:** ensiling, biomass, field margins, buffer strips, preservation success, substrate composition, fermentation pattern, biomethanisation

## Abstract

Flower strips are grown to an increasing degree in order to enhance the ecological value of agricultural landscapes. Depending on their profitable life span and the crop sequence, the strips’ biomass must be mulched after flowering to enable repeated tillage. A promising alternative is the use of the flower strips’ biomass as a co-substrate for biomethanisation – thereby contributing to the climate-friendly generation of energy. This potential bioenergy substrate occurs only seasonally and is commonly produced only in limited quantities at a farm scale. To realize the additional benefit of flower strips as energy suppliers, stock piling of the strips’ biomass is required. However, information about the ensilability of flower strip biomass is still rare. We conducted a 2-year study to analyze the ensilability of pure biomass from effloresced flower strips and mixtures of flower strip biomass with 33 and 67% whole crop maize, respectively. Ensiling took place in 3 l model silos at laboratory scale after chopping the substrate. Before ensiling several chemical characteristics of the biomass stock were determined to assess the substrate’s biochemical ensilability potential (dry matter content, water-soluble carbohydrates, buffering capacity, nitrate content). The process-engineered ensiling success after 90 days was determined based on fermentation patterns. The ensilability potential of the pure flower strip substrates reached modest levels (fermentability coefficients according to Weißbach vary around the threshold of 45). Nevertheless, acceptable silage qualities were achieved under the laboratory conditions (pH ranging from 4.2 to 4.7). Compared to pure flower strip biomass, the addition of maize noticeably improved both the substrate’s biochemical ensilability potential and the quality of real fermented silage. We conclude that a mixture of 33% biomass from flower strips with 67% whole crop maize can be regarded as a recommendable ratio if proper ensiling technology is applied.

## Introduction

Two developments characterize the current situation in the agricultural sector: the increasing demand for food (Davis et al., 2016) and the growing importance of bio-based energy production (Hennig et al., 2016). Both developments are linked via their respective land requirements and are held responsible for the negative effects of intensive land use on biodiversity (Robertson et al., 2012; Tilman and Clark, 2015). To counteract these adverse tendencies and to enhance the ecological value of agricultural landscapes, buffer strips along field margins (Mante and Gerowitt, 2006; Fritch et al., 2011) and vulnerable waterbody zones (Buckley et al., 2012) are growing in importance. For ecological and esthetic reasons, these buffer strips mostly contain a broad mixture of flowering annuals, biennials (Jacot et al., 2007) and perennials (Carlsson et al., 2017). In Europe, the support measures under the so-called second pillar of the EU Common Agricultural Policy (CAP) framework have led to a significantly increasing area of flowering strips in many regions (Haaland et al., 2011) recently.

Depending on their profitable life span and the crop sequence in which they are integrated, the strips’ biomass must be mulched after flowering in late summer in order to enable repeated tillage in early autumn. Since many species, such as mallows, can form enormous biomasses, mulching, and tilling are associated with a great deal of effort. A promising alternative is the use of the flower strips’ biomass as a source of renewable bioenergy (Christen and Dalgaard, 2013; Golkowska et al., 2016). This kind of biomass is especially appreciated as it does not compete with food production (Dauber et al., 2012; Gelfand et al., 2013) and has numerous ecological benefits, e.g., providing habitats for insects and birds. Although other conversion routes of tall herb biomass to energy like combustion (Ciesielczuk et al., 2016) are conceivable, biomethanisation is of the greatest importance (van Meerbeek et al., 2015). This technology does not require expensive drying and is most widespread in European rural areas (Capodaglio et al., 2016).

At farm scale, the biomass from effloresced flower strips crops up only seasonally and in limited quantities (Ferrarini et al., 2017). Therefore, stock piling is required if the strips’ biomass is supposed to be used as a substrate for the production of bioenergy. A well-founded knowledge of the storage capability of the biomass is essential for several reasons: (1) to avoid energy losses (Einfalt, 2017; Towey et al., 2019), (2) to prevent the entry of substances that interfere with the conversion processes, e.g., ammonia N (Poggi-Varaldo et al., 1997), (3) to make targeted use of the advantages of any preliminary conversion effects, e.g., ensiling as methane potential booster before anaerobic digestion (Teixeira Franco et al., 2016), caused by degradation processes and an increase in volatile fatty acids (VFA) (Corno et al., 2016), and thus, to design an economically efficient storage process. Expertise in the storage capability of flower strip biomass would not only be useful for the ensiling and energetic use of the flower strips, but also for harvests from perennial wild flower stands, as found in increasing numbers in restoration projects across Europe (von Cossel and Lewandowski, 2016) and North America (Voigt et al., 2012).

However, information about the ensilability of flower strip biomass is still rare. Despite an extensive literature research, only one peer-reviewed source (Oh et al., 2010) on the topic could be found. Further information stems from gray literature such as conference contributions and non-peer-reviewed technical contributions, e.g., Kalzendorf (2011). In addition, a wide range of possible seed mixtures and varieties makes it actually impossible to assume a generalizable composition of the flower strip biomass and thus, of the substrate for ensiling. Multispecies mixtures containing effloresced dicots that were neither bred nor intended for the purpose of biomass utilization and stock piling may hold some surprises regarding their carbohydrate composition, their secondary metabolites, their epiphytic population and further factors that potentially influence the ensiling success significantly.

Against the background of scarce knowledge, it seemed reasonable to determine the ensilability of effloresced flower strip biomass using an approach based on the biochemical characteristics of the biomass stock. From the substrate properties of the flower strip substrates, we intended to calculate estimates of their ensiling capability based on known biochemical principles of fermentation and to check these estimates in laboratory experiments. With this approach, we aimed for conclusions that potentially could be applied to ensiling of a wide range of wild flower substrates.

In detail, we wanted to answer the following questions:

i. What are the substrate characteristics of the biomass from effloresced flower strips? Are there peculiarities compared to well-known forage substrates?
ii. Does the standing year play a role in the substrate characteristics?
iii. How to evaluate the substrate characteristics with regard to ensilability?
iv. Are the results of characteristic-based ensilability assessments reflected by measured qualities of corresponding silages?
v. Is a mix of flower strip biomass with whole crop maize a contribution to the ensiling success?

## Materials and Methods

### Substrates

The flower mix substrates originated from plots of a field trial in Rostock (Germany, 54°04′04.1″ N 12°04′55.7″ E). The perennial flower mixture used, “BG 70” (Saaten Zeller GmbH & Co. KG), was developed especially for the use as biomass substrate in biogas plants and contains 23 species. The first sowing took place in 2014. In 2015, the experiment was repeatedly established at the same location. In this way, comparable variants could be sampled in 2015 both from the first and second main standing year after establishment. The mixed flower stands received no fertilizer. Further details on the field experiment, the seed mixture and their botanical development are given in (de Mol et al., 2018).

The growths from the effloresced flower mixture were mowed with a Haldrup parcel harvester on September 12, 2014 (first standing year after establishment) and September 16, 2015 (first and second standing years) at a stubble height of 8–10 cm. With increasing population age, we observed the tendency of the dominance of individual competing species such as melilot (*Melilotus* ssp.). Since melilot is recommended as a biogas substrate (Bull, 2014), we included a representative of the genus *Melilotus*, yellow sweet clover (*Melilotus officinalis*), in our investigation in 2015. Nearly pure stands of yellow sweet clover from field plots of the same project in Malchow (Germany, 53°59′08.8″ N 11°28′22.1″ E) were used for this purpose on September 18, 2015.

Immediately before the harvest, the yield shares of the main species components were estimated. The estimates were validated using three subsamples per variant, divided by species and weighed separately. The degree of senescence was also estimated and validated in the same way. Botanical compositions and selected field characteristics of the evaluated substrate variants are shown in Table 1.

**TABLE 1:** Main species composition and field characteristics of the flowering mixture’s substrate stocks to be ensiled.

**Substrate**	**Standing age (year of harvest)**	**Main species**	**Percentage share %**	**Senescent biomass in % FM**	**Harvest DM content in % FM**
Flower mix	1 (2014)	*Chenopodium album*	26	14.2	40.2 (1.90)
		*Malva* ssp.	24		
		*Tanacetum vulgare*	17		
		*Artemisia vulgaris*	13		
		Other species	20		
Flower mix	1 (2015)	*Malva* ssp.	28	16.8	42.1 (6.19)
		*Chenopodium album*	23		
		*Tanacetum vulgare*	18		
		*Centaurea nigra*	13		
		Other species	18		
Flower mix	2 (2015)	*Tanacetum vulgare*	23	17.8	42.8 (3.40)
		*Artemisia vulgaris*	20		
		*Malva* ssp.	18		
		*Melilotus* ssp.	16		
		Other species	23		
Yellow sweet clover	2 (2015)	*Melilotus officinalis*	99	0.5	25.0 (0.46)

*Dry matter (DM) contents are presented as means with standard deviation of the mean in brackets.*

As a reference substrate, fresh chopped whole crop maize from a neighboring field (variety “Ronaldinho,” breeder KWS^®^) harvested at early silage ripening stage was used. With the help of the BBCH scale (Weber and Bleiholder, 1990), the harvest stages were specified in terms of developmental physiology to BBCH 82 in 2014 and BBCH 87 in 2015. The maize biomass was used to prepare different mixtures with the flower strip biomass for ensiling (see section “Ensiling Procedure”).

### Ensiling Procedure

The harvested biomasses from the flower strip mixtures and from the yellow sweet clover were chopped to a length of 2–4 cm. The chopping length of the whole crop maize was 0.5–1.5 cm. All substrates were used for ensiling as pure substrates (100% flower mix substrate = FM100; 100% maize = ZM100; 99% yellow sweet clover = YSC99; see Table 1). In addition, mixed substrates from the flower strip’s biomasses with maize were prepared. The mixing ratios were 1:2 (33% flower mix, 67% maize = FM33) and 2:1 (67% flower mix, 33% maize = FM67). Proportions are based on fresh weights immediately before ensiling. In terms of dry matter, this would correspond to a flower biomass:maize – mixing ratio of 2.9:1 in 2014 and 3.6:1 in 2015 for FM67, and a ratio of 0.7:1 (2014) and 0.9:1 (2015) for FM33, respectively.

The feedstock substrates were ensiled in at least three replicates in 3 l glass jars. The jars were washed and sterilized (180°C, 8 h) before the substrates were filled in and compressed in layers by hand. The resulting final packing densities ranged from 0.35 to 0.60 g cm^–3^ DM. The filled jars were closed air-tight with a rubber-lined lid that was fixed by clips. Glass jars of all treatments were stored in a dark, tempered room (16°C) for 90 days. After ensiling the silages were removed from the glass jars, sealed airtight in plastic bags and stored at −40°C prior to the analyses of fermentation profiles.

Furthermore, subsamples from each substrate (ca. 500 g FM) were dried in a temperature-controlled range of <45°C and thereafter grounded to a sieve mesh of 1 mm wide. The four field repetitions were reduced to two test repetitions for lab capacity reasons using a sample splitter. This pooled material was used for the determination of the substrate’s biochemical properties in both test years.

### Biochemical Analyses

Several biochemical parameters which are suitable to estimate the ensilability and the fermentation success were determined from the substrates immediately before ensiling and from the fermented substrates after ensiling, respectively. In the study period 2015, the analysis spectrum could be extended to nitrate, buffering capacity (BC) and NDF (see subsection “Parameters Characterizing Substrate’s Ensilability”).

#### Parameters Characterizing Substrate’s Ensilability

DM content of the feedstock immediately before ensiling was determined by oven drying at 45°C to a constant weight. BC was analyzed by titration with lactic acid (0.1 mol l^–1^) to a pH of 4.0 according to (Weißbach, 1992). We analyzed the sum of water-soluble carbohydrates (WSCH) and the enzyme-insoluble organic matter (EULOS) by Near Infrared Reflectance Spectroscopy (NIRS, Bruker^®^ MPA, Bruker, Germany) with the photometrical Anthron method according to Naumann and Bassler (2012) as the reference for WSCH and the enzymatic method according to de Boever (de Boever et al., 1986) as the reference for EULOS. Dry combustion technique (Elementar^®^ Analyzer, Vario Max CNS, Elementar, Germany) has been adapted to determine crude protein contents (CP, N × 6.25). Nitrates were analyzed by continuous-flow analysis (CFA Analyzer AA3, Seal^®^, Germany). Neutral detergent fibre (NDF), acid detergent fibre (ADF), and crude fibre (CF) were determined by wet chemical analyses using a Fibretherm, Gerhardt^®^, Germany. Hemicellulose contents have been estimated as the difference between NDF and ADF concentrations.

In order to characterize fermentability in a more holistic manner, the two parameters DM and WSCH/BC were combined to the fermentability coefficient (FC) according to Weißbach and Honig (1996):

(1) FC=DM[%]+8WSCH/BC

Feedstocks with FC < 35 are considered as “difficult-to-ensile,” whereas those with FC > 45 are referred to as “easy-to-ensile.”

#### Fermentation Characteristics of Ensiled Substrates

After thawing of the frozen silage samples at room temperature, silage extracts were prepared from 50 g silage and 200 mL deionized water. The pH values of these extracts were measured potentiometrically by a calibrated pH analyzer (precision 0.01). Between each measurement of pH, a cleaning of the probe was carried out with distilled water. Fermentation products were analyzed in the filtrated extracts thereafter. Lactic acid was determined by HPLC (Aminex HPX-87H, Bio-rad^®^, United States) with a flow rate of 0.60 ml min^–1^ at the UV detector. Short-chain fatty acids and ethanol were quantitatively separated by gas chromatography (GC-14A, CLASS-VP, Shimadzu^®^, Kyoto, Japan). The ammonium content in the silage extracts was determined according to the method of Voigt and Steger (1967). Silage DM was determined by drying to a constant weight (105°C, 24 h) and was corrected for the loss of volatiles during drying as described by Weißbach and Strubelt (2008a, b). Ashing followed after drying at 600°C for at least 4 h in a muffle furnace until obtaining a light gray ash color and led to the parameter crude ash content (CA).

#### Potential Biogas Yield Estimation

The potential for methane formation was estimated using practice-proven estimation equations based on biochemical parameters of the substrates before ensiling (Weißbach, 2009).

(2) $$\mathrm{ZM100}:V\,S=984-\left(C\,A\right)-0.47\,\left(C\,F\right)-0.00104\,{\left(C\,F\right)}^{2}$$

(3) FM100,YSC99:V⁢S=1000-(C⁢A)-0.62⁢(E⁢U⁢L⁢O⁢S)

$$-0.000221\,{\left(E\,U\,L\,O\,S\right)}^{2}$$

The substrate’s amount of fermentable organic substances (VS g kg**^–^**^1^ DM) was estimated for pure maize using Eq. (2) and for all other pure substrates using Eq. (3). Mixed substrates were assessed by weighted means of (2) and (3) according to the mass proportion of the single substrates.

Substrate-specific biogas (BGY) and methane (CH_4_Y) yield potentials of the tested feedstock substrates were derived from VS as follows:

(4) B⁢G⁢Y=0.80⁢(V⁢S)

(5) $$C\,H_{4}\,Y=0.42\,\left(V\,S\right)$$

BGY and CH_4_Y are given in norm liter per kg (NI kg DM**^–^**^1^) and are corrected of VFA.

### Data Analysis

Biochemical composition data are presented as averages and standard deviation of the mean (*s*_*d*_) with *n* = 2 replicates. In the absence of real local repetitions, the effects of the standing age on substrate’s biochemical properties were analyzed including the flower-maize-mixtures FM67 and FM33 as replicates. The parameters whose values were below the detection limit (“not detected”) in most samples were not included in studying the differences in the biochemical compositions of the silages.

All evaluation-relevant data records were first tested for normal distribution using the Shapiro–Wilk test. For a given normal distribution, analysis of variance (ANOVA) was applied to investigate the effects of the factors “substrate” (2014 and 2015) and “standing age” (2015 only). If the values were not normally distributed and neither log nor sqrt transformations achieved a normal distribution, mixed linear models were applied with “substrate” and “standing age” as a fixed factors and “year” as random variable. Modeled parameters were estimated with an ANOVA of type III and a Satterthwaite’s adjustment.

The substrate specific patterns of the fermentation products were visualized with non-metric multidimensional scaling (NMDS) based on Bray–Curtis distances. The influence of substrate properties on fermentation profile was additionally tested with a goodness-of-fit permutation test using the squared correlation coefficient as test statistics.

All statistical analyses were performed by scripts using the R environment version 3.3.2 (R Development Core Team, 2016). The R-package “lme4” was used to calculate the mixed linear models (Bates et al., 2015), and the “vegan” package to perform NMDS (Oksanen et al., 2018).

## Results

### Substrates’ Biochemical Properties

The substrates’ properties with a known or reasonably suspected influence on the ensiling capability were determined from substrates immediately before ensiling in 2015 (Table 2). With more than 40%, the dry matter content was highest in the pure flower strip mixture substrate (FM100). The lowest DM content was found in the silage maize, which was not yet fully silage-ripened. The blends of flower strip mixture and maize reached intermediate values. No trend in DM content could be discerned with regard to the feedstocks of different standing ages. The ash contents of the flower mixture substrates were very low (<7% DM). However, it should be noted that the mixtures were harvested using plot technology.

**TABLE 2:** Chemical characterization of the tested feedstock variants before ensiling (experimental year 2015, means from two laboratory repetitions with standard deviations in parentheses).

**Type of feedstock substrate^1^**	**FM 100**		**FM 67**		**FM 33**		**ZM 100**	**YSC 99**
**Standing year**	**1**	**2**	**1**	**2**	**1**	**2**	**1**	**1**
**Parameter^2^**								
Dry matter content (g kg^–1^)	426.7 (8.0)	400.9 (1.1)	363.8 (6.4)	380.7 (2.2)	325.5 (2.2)	315.0 (1.9)	266.6 (3.8)	268.6 (4.0)
Crude ash (g kg^–1^ DM)	63.8 (1.7)	65.3 (0.9)	66.0 (1.7)	62.0 (0.5)	53.8 (0.4)	52.0 (1.2)	32.6 (0.4)	89.3 (0.8)
Crude protein (g kg^–1^ DM)	61.1 (6.3)	55.3 (3.9)	69.2 (4.2)	69.0 (2.2)	75.2 (0.4)	81.2 (2.5)	74.1 (11.2)	213.7 (5.1)
Crude fiber (g kg^–1^ DM)	460.7 (39.9)	426.6 (23.7)	399.7 (25.6)	388.1 (49.3)	330.3 (50.1)	282.2 (50.2)	222.5 (4.4)	242.9 (13.0)
Hemicellulose (NDF-ADF, g kg^–1^ DM)	160.6 (4.1)	215.4 (2.5)	184.9 (1.3)	215.5 (1.8)	182.2 (2.5)	187.0 (1.8)	215.2 (2.7)	110.6 (1.6)
Water-soluble carbohydrates (g kg^–1^ DM)	3.2 (0.6)	9.9 (0.3)	15.1 (0.4)	36.1 (0.2)	85.1 (0.3)	115.5 (3.4)	182.9 (1.5)	49.9 (0.8)
Nitrate content (g kg^–1^ DM)	1.5 (0.01)	0.1 (0.06)	0.7 (0.21)	0.1 (0.05)	0.6 (0.16)	0.2 (0.07)	0.3 (0.04)	0.3 (0.05)
Buffering capacity (g LA kg^–1^ DM)	9.2 (0.14)	6.8 (0.10)	8.5 (0.11)	8.0 (0.01)	6.8 (0.16)	6.9 (0.05)	10.4 (0.65)	22.4 (0.28)

*^1^FM100 – 100% flower mix; FM67 – 67% flower mix, 33% maize; FM33 – 33% flower mix, 67% maize; ZM100 – 100% maize; YSC99 – 99% yellow sweet clover. ^2^DM, dry matter; LA, lactic acid; WSCH, water-soluble carbohydrates.*

Pure substrate from effloresced flower mixtures were characterized by very high crude fiber contents (>45% DM) and low crude protein contents (<8% DM) reflecting the late ontogenetic state of the dominating plant species at the harvest time. In contrast to this, yellow sweet clover (YSC99), which has the potential to dominate perennial flower strips after several years of use, had CF (25.6% DM) and CP (22% DM) values that resemble legume forage plants such as alfalfa. The nitrate content was the only characteristic that has been significantly influenced by the age of the flower strips stand (*F* = 7.78; *P* = 0.049). In the second year after the establishment of the mixtures, the nitrate content of the harvested biomass decreased by 1.4 to only 0.1 g per kg DM. A tendency toward higher WSCH values could not be statistically confirmed.

### Substrates’ Ensilability Assessment

**FIGURE 1:**
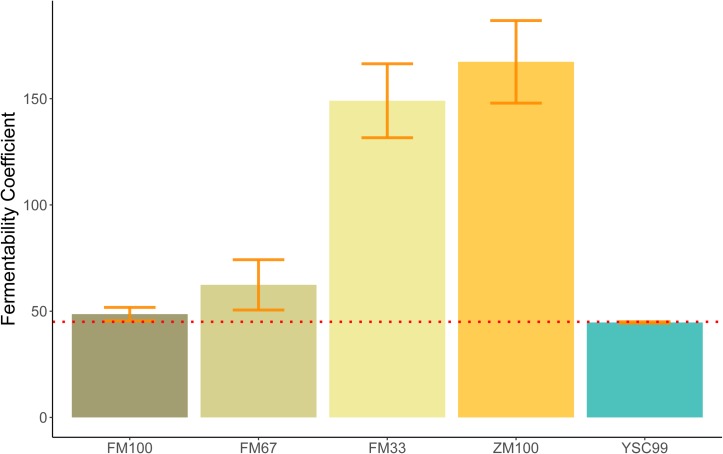
Mean Fermentability Coefficients (FC) of the tested feedstock substrates. Error bars indicate standard deviations of the mean. (Sample size: FM100, FM67, FM33 *n* = 4; ZM100, YSC99 *n* = 2). The red dotted line indicates the FC threshold according to Weißbach and Honig (1996).

The FC of the pure flower mix substrate was not significantly influenced by its standing age (*F* = 0.216; *P* = 0.666). This fact allowed us to average the FC values over the levels of this factor (Figure 1) and find a significant effect of the substrate type on the FC (ANOVA, *F* = 17.98; *P* = 0.020^∗^).

**FIGURE 2:**
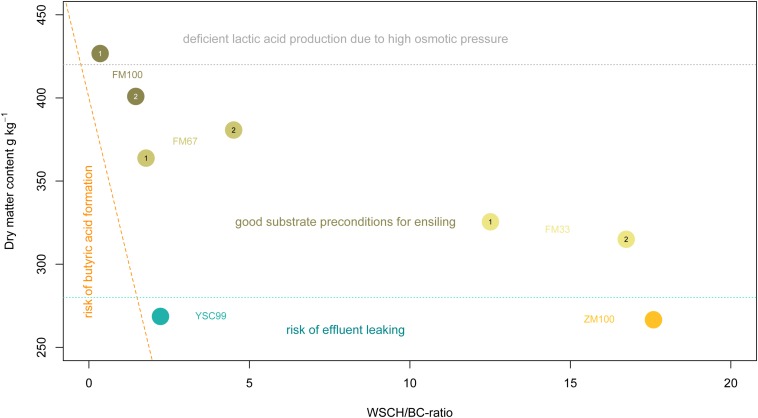
Arrangement of the tested feedstocks in the estimation frame according to Weißbach and Honig (1996). The digits 1 and 2 within the location points indicate the standing year. The dashed orange line reflects the critical dry matter content as a function of the WSCH/BC-ratio. The dotted light-gray line shows approximately the beginning of the range of limited metabolic activity of natural epiphytic lactic acid bacteria population due to forced osmotic pressure.

The substrate-specific characteristics of FC in the study are shown in Figure 1. All substrates containing maize as a component clearly exceeded the FC > 45 threshold and thus indicate good conditions for low-loss preservation. The unusually high values of the maize-dominated test variants FM33 and ZM100 are due to their very high content of WSCH, which is also reflected in high WSCH/BC-ratios (see also Figure 2). In contrast to mixtures with maize, the two pure flower mix substrates FM100 and YSC99 had FCs that are within the limits of good conservation suitability.

Below 28% DM, an increasing risk of leachate from the feedstock must be expected. However, the effloresced stands had sufficiently high (>30%) contents of DM without wilting efforts (Figure 2). This finding does not apply to the dominant stocks of yellow sweet clover (YSC99) whose biomass was still vital at the time of harvest and contained little senescent material. On the other hand, dry matter contents of the pure flower mixture in the first cropping year exceeded the recommended DM-range of 30–40% and reached a level that is only suited as a metabolic substrate for very osmotolerant lactic acid producers.

### Realized Silage Quality

Silage fermentation patterns varied according to substrate, year, and standing age. ANOVA after fitting GLMM models revealed significant effects of substrate types on silage characteristics for most of the main fermentation products (Table 3), namely pH, lactic acid, acetic acid, and ethanol. The only exceptions were butyric acid and propionic acid, since their contents were partly below the detectability threshold and thus escaped the biostatistical model estimations.

**TABLE 3:** Main fermentation products of the tested lab-scale ensiled feedstock after a storage period of 90 days (2 year means with standard deviations in parentheses).

**Feedstock Substrate^1^**	**Standing age (years)**	**pH**	**Lactic acid (g kg^–1^ DM)**	**Acetic acid (g kg**^–^**^1^ DM)**	**Butyric acid (g kg**^–^**^1^ DM)**	**Propionic acid (g kg**^–^**^1^ DM)**	**Ethanol (g kg**^–^**^1^ DM)**
FM100	1	4.54 (0.09)	4.57 (1.69)	1.45 (0.23)	0.62 (0.217)	0.09 (0.006)	0.38 (0.17)
	2	4.30 (0.24)	4.09 (0.84)	1.07 (0.12)	0.02 (0.003)	0.04 (0.004)	0.11 (0.05)
FM67	1	3.92 (0.06)	6.22 (0.53)	1.57 (0.29)	n.d.	0.01 (0.002)	0.75 (0.55)
	2	3.86 (0.02)	6.69(0.32	1.34 (0.24)	n.d.	0.01 (0.002)	0.70 (0.26)
FM33	1	3.90 (0.23)	6.60 (0.32)	1.80 (0.49)	n.d.	n.d.	0.51 (0.07)
	2	3.72 (0.02)	6.84 (0.21)	2.30 (0.44)	n.d.	n.d.	0.64 (0.06)
ZM100	1	3.64 (0.09)	7.80 (0.43)	2.22 (0.14)	n.d.	0.12 (0.015)	1.43 (0.44)
YSC99	1	4.61 (0.03)	9.66 (1.27)	1.88 (0.32)	n.d.	0.03(*n*.*f*.)	1.25 (0.25)
**ANOVA results (*F*; *P*)**							
	Substrate	*F* = 112.69; *P* < 0.001	*F* = 33.81; *P* < 0.01	*F* = 10.24; *P* < 0.01	n.f.	n.f.	*F* = 19.09; *P* < 0.01
	Standing age	*F* = 3.90; *P* = 0.055	*F* = 0.59; *P* = 0.446	*F* = 0.28; *P* = 0.599	n.f.	n.f.	*F* = 0.17; *P* = 0.684

*n.d., not detectable; n.f., not feasible. ^1^FM100 – 100% flower mix; FM67 – 67% flower mix, 33% maize; FM33 – 33% flower mix, 67% maize; ZM100 – 100% maize; YSC99 – 99% yellow sweet clover.*

Despite trends in feedstock analysis before ensiling (see “Substrates’ Ensilability Assessment”), standing age caused only minor variation in the main silage characteristics leading to non-significant effects in the mixed models.

**FIGURE 3:**
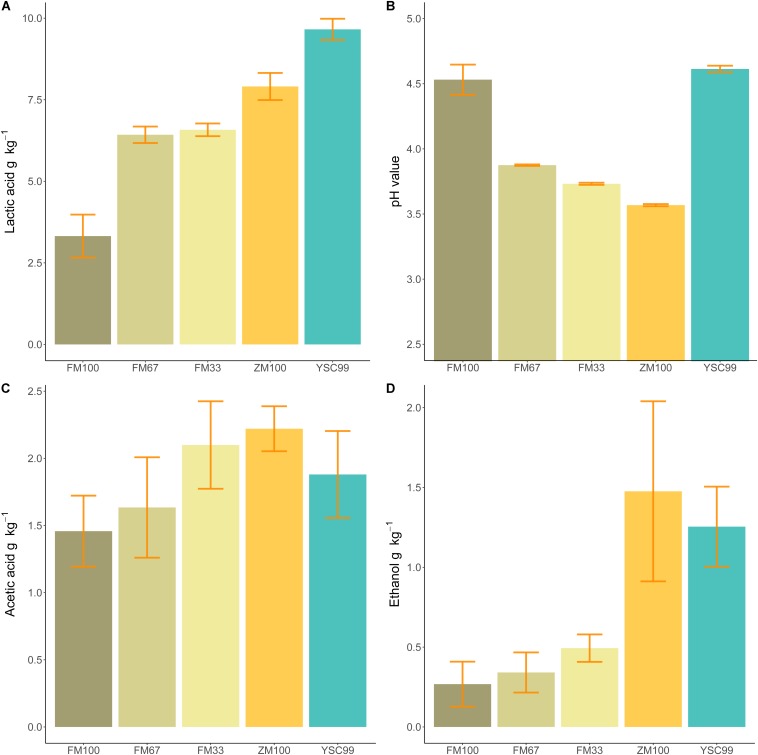
Mean fermentation products of the tested feedstock substrates in 2015. **(A)** Lactic acid content, **(B)** pH value, **(C)** acetic acid content, **(D)** ethanol content. Error bars indicate standard deviations of the mean (Sample size: FM100 *n* = 4, FM67 *n* = 4, FM33 *n* = 5; ZM100 *n* = 5, YSC99 *n* = 3).

Only lab-silages containing maize fell below the pH value threshold of four (Table 3). Undesirable butyric acid was found only in the variants of the pure flower mix substrates with DM contents of more than 40% in the harvested substrate. In order to allow a better comparison of the silage with the properties of the harvested substrate, which was investigated only in 2015, relevant fermentation parameters of the results from 2015 are shown separately in Figure 3.

When comparing the amount of lactic acid formed (Figure 3A) with the corresponding pH values (Figure 3B), it is noticeable that yellow sweet clover did not follow the common trend of decreasing pH values at higher lactic acid concentrations. Since acetic acid and ethanol are metabolites of the same bacterial group (coli-erogenic), their contents in the laboratory silos were compared (Figures 3C,D). The comparison revealed that during ensiling of effloresced flower mixture biomass, less alcohol was formed in relation to acetic acid.

### Relationship Between Substrate Properties and Fermentation Profiles

**FIGURE 4:**
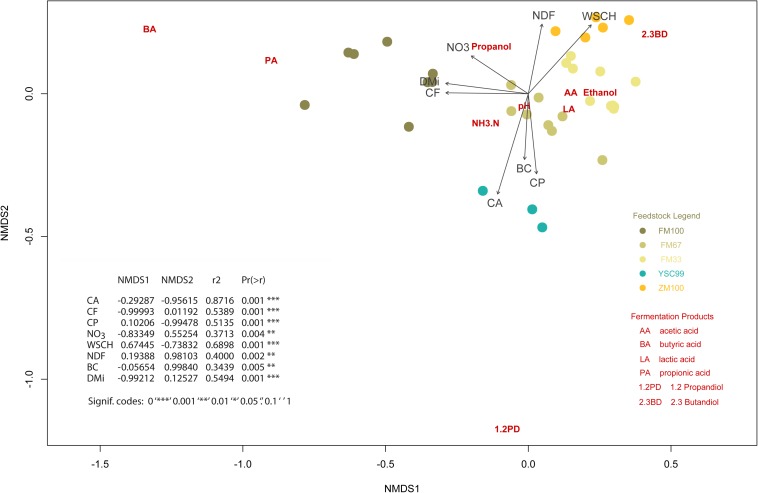
Non-metric multidimensional scaling ordination plot showing the position of the fermentation characteristics (dark red colored abbreviations) in relation to the initial biochemical substrate properties (darkgray colored arrows including abbreviations). The location of the corresponding substrates is additionally point-plotted and explained in a legend. Nomenclature of the biochemical characteristics: CA, crude ash; CF, crude fiber; CP, crude protein; NDF, neutral detergent fiber; NO_3_, nitrate; WSCH, water soluble carbohydrates; BC, buffering capacity; DM_i_, initial dry matter content of the substrates (before ensiling).

In order to make relationships between substrate biochemical characteristics and fermentation patterns visible, a complex multivariate analysis was carried out. We applied a NMDS which allowed us to include the whole range of characteristics in the analysis and to represent them graphically (Figure 4). The goodness of fitting the multidimensional data to the reduced dimensioned NMDS was god (see Supplementary Figure A1 for details).

The plot contains a table presenting the results of the vector fitting procedure additionally. The data on the expression of the substrate characteristics before ensiling served as vectors. The substrate characteristics of this figure-integrated tabular list were arranged according to the closeness to the matrix of fermentation characteristics, expressed by the squared correlation coefficient. These are also the vectors with a relatively high gradient length, which can be seen from the length of the arrows.

On the one hand, it is noticeable that the individual substrates always form well-defined clusters if they are 1-year stocks. On the other hand, there is a trend toward splitting into subgroups, as in the case of the pure flower mixture variant FM100, shown on the left side of the plot.

## Discussion

### Substrate Characteristics and Fermentation Patterns

To our knowledge, this study is the first exploring the ensilability of effloresced flower strip’s biomass. Regarding the scarcity of data concerning biomasses from wildflower mixtures, we consider the description of the substrate characteristics valuable as well; especially since the botanical composition of the stock is known and adequately described. With the inclusion of melilot, the 2-year study shows quite a wide range of possible substrate compositions despite limited numbers of variants.

The high fiber contents found in the growths of the flowering strips together with the high percentages of senescent foliage, low protein and sugar contents are characteristics of fast-growing, high-flowering dicotyledons with a low tendency to vegetative regeneration and persistence. Such substrate constellations offer poor conditions for successful ensiling due to a lack of readily available sugars for the lactic acid formation (Pitt, 1990) and a high stock of harmful molds and yeasts (Dunière et al., 2013). Consequently, a low lactic acid content of only 4 g kg^–1^ was formed in the pure flowering mixture silage (FM100). Nevertheless, this was sufficient to lower the pH value to below 4.7, which is necessary for stable storage at a dry matter content of 40% (Kalač, 2011). The occurrence of butyric acid indicates that the reduction of the pH value was slow, so that the preservative acidification effect was not yet present in the initial storage phase. A certain contribution of fiber degradation to low molecular saccharides could also have contributed to continued lactic acid formation. Unfortunately, the fiber fractions of the silages after fermentation were not analyzed again, which could have helped to verify this thesis by comparing pre-ensiling with post-ensiling results. If we recall the ordination (Figure 4, left pointing arrows), we can see that the characteristics CF, DM, and NO_3_ have the greatest influence on the fermentation patterns of pure FM100-silages. However, it is not very likely that the contribution of crude fiber to the explanation of fermentation profiles is related to the carbohydrate donors. If that was the case the NDF arrow would rather point in the direction of the FM100 positions. Instead, it seems to be the effect of an intercorrelation with the dry matter content: the older the plants in the stand, the drier and more fibrous they become. It is therefore obvious to assume that the ontogenetic development of the flower mixture stands is the significant background-variable and responsible for variations in silage quality. Obviously, the known Clostridia-suppressive effect of nitrate (Kaiser et al., 1999) is rather important in the limit range of fermentability.

For the fermentation acid patterns of the maize-dominated silages, the height of the WSCH and the NDF fraction played an important role (Figure 4, right pointing arrows), although there was no lack of easily fermentable saccharides. Nonetheless, the ratio of the fermentation products lactic acid, acetic acid, ethanol and 2,3-butanediol might have been influenced by these ingredients in a way which has not been recognized as random.

### Substrate’s Ensilability Assessment

The prediction of ensiling success on the basis of the substrates’ biochemical properties is both a promising and a difficult undertaking, as not only biochemical, but also physical and microbiological processes are involved (Müller and Bauer, 2006). Assuming a proper ensiling technology and an average lactic acid bacteria (LAB) stocking on the phyllosphere is given, the existing estimation framework can be successfully applied for the major forage crops (Weißbach and Honig, 1996). The few authors dealing with the fermentability of herbs or herb-rich growths (Daniel and Opitz von Boberfeld, 1997) found that some of these species oppose specific effects on fermentation processes and attribute this to secondary metabolites (Weißbach, 1998). Consequently, the conservation results could not be reliably predicted with the existing substrate-based estimation frameworks. In our study, the flower strip mixtures also contained plants with notable amounts of antimicrobially active secondary metabolites like *Melilotus* (coumarins) or *Tanacetum* (flavonoids, terpenes, coumarins). Nevertheless, we can state that the results of the ensilability classifications prior to ensiling (Figures 1, 2) are sufficiently consistent with the fermentation profiles of the silages obtained from them. Therefore, our results do not argue against the application of the existing estimation frames (developed for forages) for the ensiling of flower strip mixtures. However, one should be aware that particularly high concentrations of antimicrobial active metabolites, similar to variations in nitrate contents in the feedstock, could modify the ensiling success. In order to expand the still rare knowledge in this respect, further targeted investigations are necessary, both on the laboratory level and in practice.

### The Effect of Standing Age on Ensilability of Biomass From Flowering Strips

In our analyses, the factor “standing age” proved to be of little influence on ensilability. However, this was also partly due to differences in the degrees of freedom (one degree for factor “standing age” against five degrees for the factor “substrate”) and thus, due to the study design. The short rotation type of flower stripe examined here represent the most frequently occurring option of buffers in European arable landscapes due to designated support schemes and administrative regulations. The effect of the year of use on ensilability has two aspects: the changes in the soil nutrient pool and the botanical shifts in the mixed stands. In the comparison of the first year with the second standing year, both processes left their imprints on the biochemical characteristics of the grown substrates. The significant decrease in the nitrate content is a sign of N-limitation that is already beginning in the second year after establishment. Although there were no serious shifts in the abundance of the dominating species, higher contents of WSCH and lower CF concentrations indicate physiologically younger plant material in the second standing year. This finding could also be explained by more restrained growth due to N-depletion. From the point of view of ensilability assessment, this results in advantages for the availability of monosaccharides for lactic acid formation, but also in disadvantages for butyric acid inhibition with increasing standing age. According to Kaiser et al. (1997), a minimum content of 1.5% NO_3_ should be targeted in order to achieve sufficient safety against butyric acid formation under field conditions. In our experiment, the advantages and disadvantages of the age-affected substrate pattern apparently compensated each other, so that there were no significant deviations with regard to the fermentation profiles.

In the case of perennial flowering mixtures, experience has shown that the age of the crop stand can have a major influence on substrate characteristics, especially if there is a stronger shift from annuals to perennials (de Cauwer et al., 2006). Under humid climate conditions, grass coverage increases with increasing standing age (de Cauwer et al., 2005). This stand development can lead to an improvement in ensilability if at least two cuts are made. However, this development reduces many ecosystem services of flowering strips. In addition, nitrogen fertilization would also be required to maintain a level of biomass production, which justifies mowing and transport.

### Further Implications for the Storage of Biomass From Flowering Strips

In accordance with Teixeira Franco et al. (2016), we consider classical measures of production engineering measures such as short chopping lengths and good compaction to be more important than additives in order to ensure a low-loss storage of biomass – also from flower mixtures designed for energy recovery. However, the use of additives to increase storage safety or energy yield (Herrmann et al., 2011) is widespread. Based on our investigations of the substrate composition, it seems that if an application of additives was considered for late harvested flower strips, enzyme application would be more promising than inoculation with LAB. Generally, late summer growths have a high content of natural epiphytes (Filya et al., 2007), including LAB, so that LAB-inoculations are not necessary to guarantee the desired lactic acid formation. In fact, there is a risk that the inoculant LAB are overwhelmed by the natural epiphytes and do not affect fermentation significantly (Muck, 1989). Moreover, contents of less than one percent WSCH are not sufficient for an economically justifiable LAB-application (Bolsen et al., 1996). On the other hand, hemicellulose contents up to 21% DM are a promising pool for successful depolymerization by suitable enzyme products (Schimpf et al., 2013) that have the potential to enhance biogas yield if biomethanisation was chosen as conversion path for biomass from flower stripes.

Gravimetrically determined mass losses of laboratory silos like jars are not really suitable to describe the storage losses of biomass to be expected under real conditions of a field storage pile (Wendt et al., 2018). The individual weighings carried out as part of our study showed losses on the order of 0.5% and essentially reflected the fermentation activity as a whole. The latter, in turn, is strongly dependent on the DM content. Therefore, approaches such as those of Goeser et al. (2015) to draw conclusions about the expected losses under practical conditions from the fermentation patterns appear more successful. Following this logic, the mixture FM33 has to be recommended, since the proportion of fresh maize is sufficient to form an adequate amount of lactic acid for butyric acid-free storage, but the advantage of the higher dry matter content from the flowering strip biomass – another precondition of low storage loss – is still evident.

### Technological Aspects of Realizing the Bioenergy Potential of Biomass From Flowering Strips

For the energetic utilization of biomasses rich in lignocellulose, such as that of flower strips, a number of conversion routes are possible, e.g., combustion (van Meerbeek et al., 2015), ethanol (Chen et al., 2011) or biogas production (Vollrath et al., 2016). For the latter two techniques, ensiling is an important component of the production process (Chen et al., 2007) facilitating storage (Emery et al., 2015) and pre-treatment of the substrate (Essien and Richard, 2018).

Economically and logistically, the way of utilization is to be preferred, that not only copes best with the substrate’s qualities but also enables short routes of transport. Regarding the routes of transport biomethanisation is the preferred way to process biomass from flowering strips in the rural areas of Europe due to the large number of decentralized biogas plants (Capodaglio et al., 2016). The question of the substrate quality, however, cannot be answered independently of the specific type of biogas plant. Certainly, very few plant operators would rely on a substrate that delivers significantly lower methane yields than maize. In the present study it became obvious that using maize as a co-substrate is essential to realize the bioenergy potential of flower strip biomass. On the one hand maize proved to be an excellent mixing substrate to ensure low-loss ensiling of the flower strip biomass, especially in the case of the variant FM33. On the other hand, the mixing with maize optimized the specific methane yield of the flower strip biomass. The pure flower mixture (variant FM100) only had a specific methane yield of approx. 180 Nl CH_4_ kg^–1^, while the mixed substrate (FM33) with 67% fresh maize content yielded approx. 300 Nl CH_4_ kg^–1^ representing nearly 90% of the reference yield of pure maize (see Supplementary Figure A2). Thus, the FM33 variant did not only have the best storage properties, but also promises high acceptance as substrate by the operators of biogas plants.

The production of a mixed substrate, however, remains a challenge at the commercial scale. The optimum crop for mixing would be maize that has not yet matured too far with DM contents of 22–28% on a whole plant basis; in particular if the biomass of the effloresced flower mixture is no longer vital and exceeds DM contents of more than 40%. The maize would supply the substrate with high contents of WSCH and moisture to lower the osmotic pressure and to enhance the compactability of the feedstock during ensiling. In practice silage maize is harvested at DM contents of 30–36%. Therefore, it may be a good idea to apply the widespread practice to use maize from the field edges and from hunting corridors as an early mixing substrate that is harvested before the actual silage maize campaign starts.

## Conclusion

The use of increasing amounts of flower strips’ biomass as a source of renewable bioenergy is a promising option to reconcile economic and environmental concerns. A primary challenge associated with the realization of this alternative is to store the feedstock in a way that losses are minimized. Due to the similarity of the biomass with delayed harvested forage, ensiling offers a cost-effective form of storage. Since there is little experience with the ensiling capability of flower strip mixture’s substrates, we studied the ensilability of botanically classified and composition-related described feedstock from late harvested flower strips as pure substrate or blended with whole-crop maize. This study showed that existing frameworks developed for roughages could be successfully applied to predict the ensiling success on the base of the substrates’ biochemical properties. This knowledge is important in order to make the right preparations and process-related decisions that lead to low-loss storage of this largely unknown feedstock. Pure biomass from effloresced flowering strips is set on a certain risk of misfermentation if not blended with a favorable feedstock like maize. We conclude that a mixture of 33% biomass from flower strips with 67% whole crop maize can be regarded as a recommendable ratio for low-loss storage. In addition, the multivariate approach used in this study to uncover the relationship between characteristics of the initial substrate and the fermentation pattern seems applicable for further investigations of substrate storage as a basis for the production of bioenergy.

## Data Availability Statement

All relevant data is contained within the manuscript. In addition, raw data from processed data will be made available by the authors, without undue reservation, to any qualified researcher on request.

## Author Contributions

JM and JH contributed to the conception and the design of the study; JH organized and administrated the project; JM wrote the first draft of the manuscript; JH supplemented and improved the manuscript; JM performed the statistical analysis in coordination with JH. Both authors contributed to manuscript revision, read and approved the submitted version.

## Conflict of Interest

The authors declare that the research was conducted in the absence of any commercial or financial relationships that could be construed as a potential conflict of interest.

## Supplementary Material

FIGURE A1 Shepard plot showing scatter around the regression between the interpoint distances in the final NMDS configuration against their original dissimilarities.

FIGURE A2 Substrate-specific biogas **(a)** and methane **(b)** yield potentials of the tested feedstock substrates calculated according to Weißbach (2009). Calculations are based on ash content, crude fiber content, and enzyme solubility of harvested substrates before ensiling. Numbers in the bar indicate the standing age of the biomass stock. Substrates nomenclature: FM100 = pure biomass from flowering stripes, FM67 = mixture of 67% flower stripe’s biomass and 33% silage maize, FM33 = 33% flower stripe’s biomass and 67% silage maize, ZM100 = pure silage maize, YSC99 = 99% yellow sweet clover.
