# Supplementary figures and images for: Ensilability of Biomass From Effloresced Flower Strips as Co-substrate in Bioenergy Production
Source: Front Bioeng Biotechnol. 2020 Jan 31;8:14. doi: 10.3389/fbioe.2020.00014 (PMC7006225; doi:10.3389/fbioe.2020.00014)

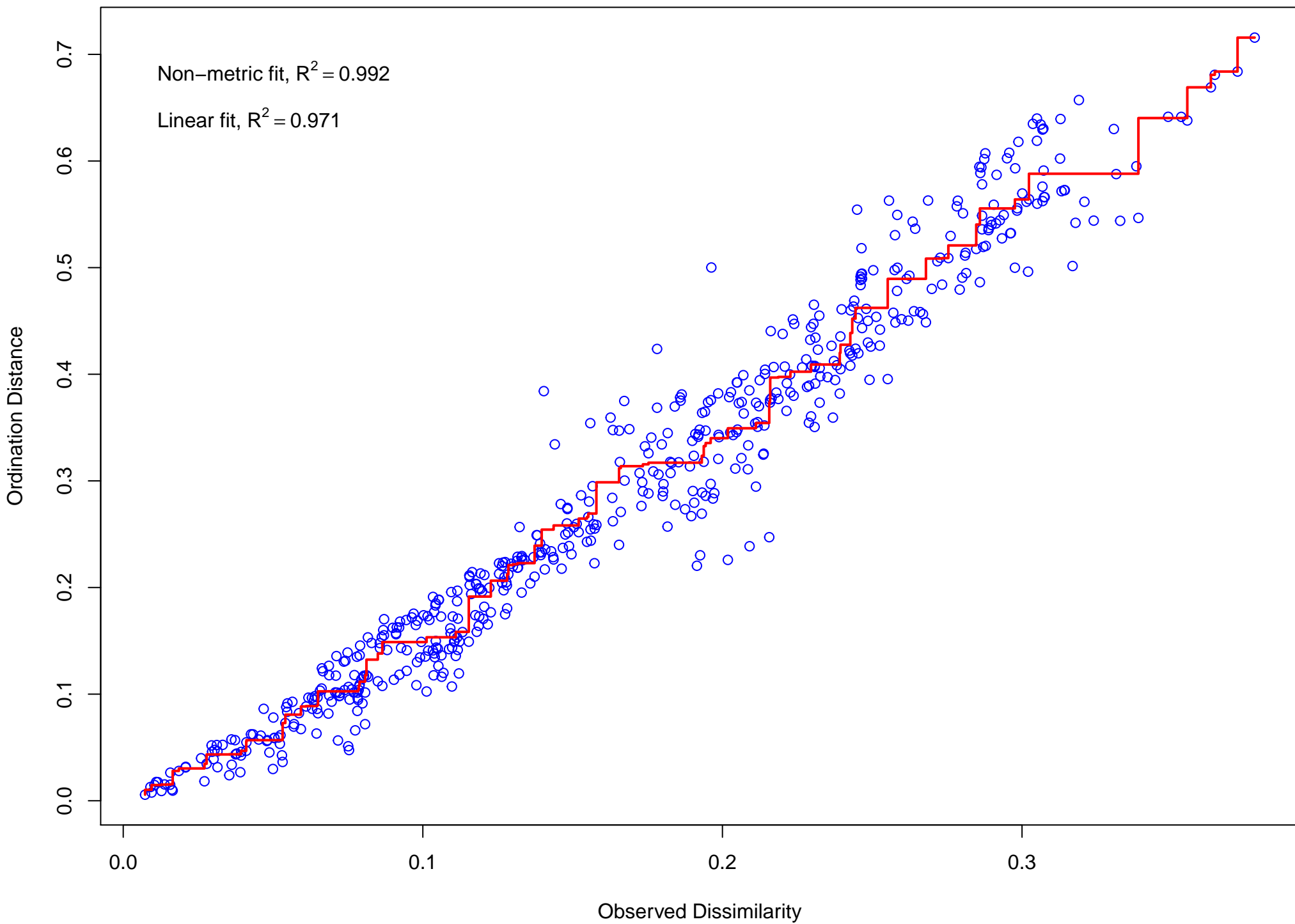

Supplement: FIGURE A1 — Shepard plot showing scatter around the regression between the interpoint distances in the final NMDS configuration against their original dissimilarities. [file Data_Sheet_1.PDF]

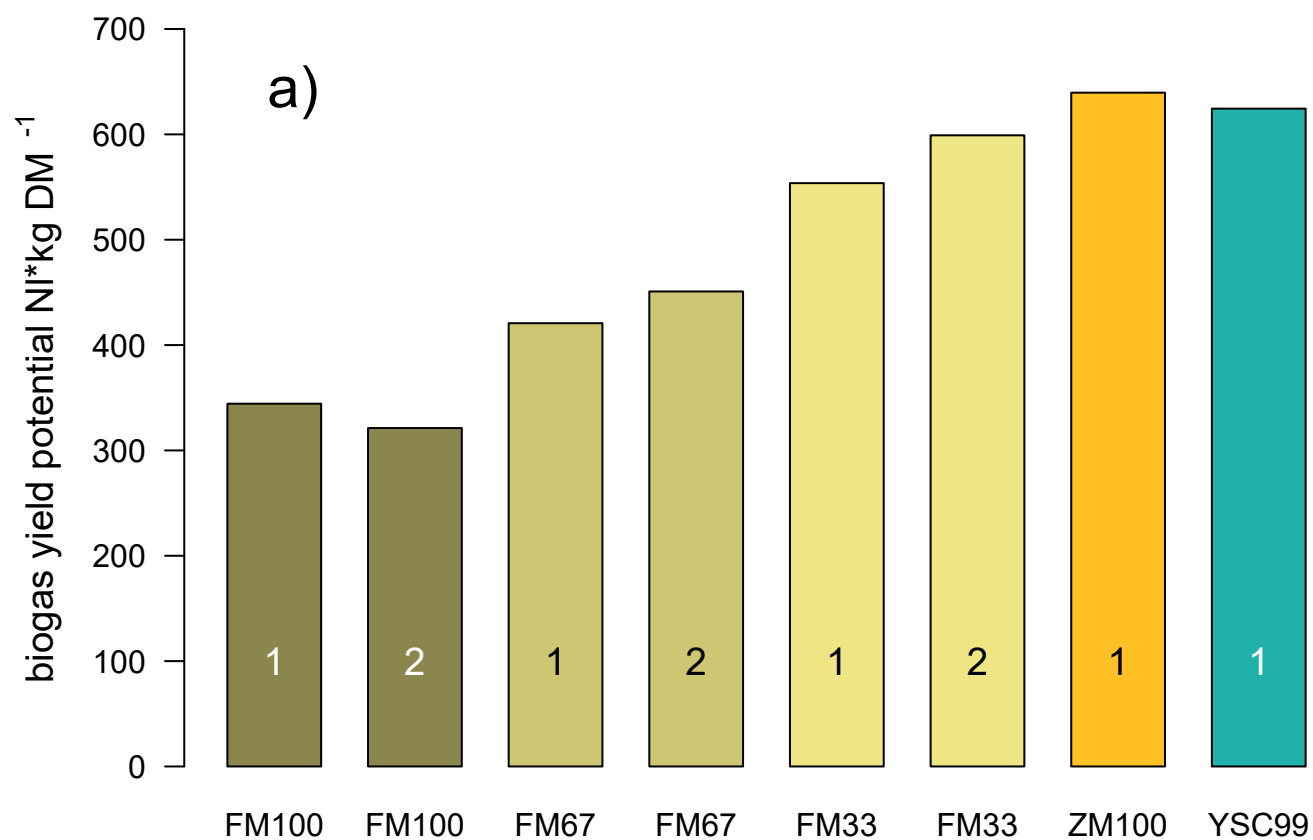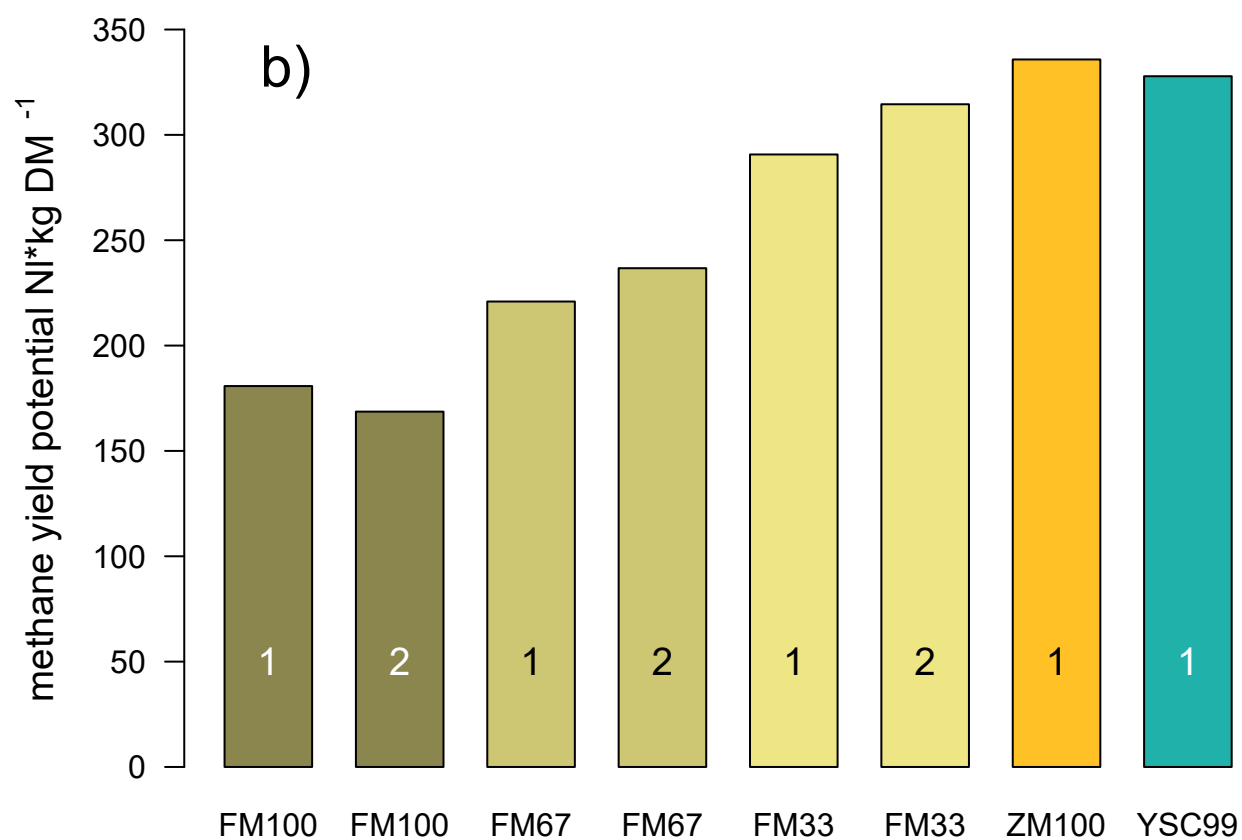

Supplement: FIGURE A2 — Substrate-specific biogas (a) and methane (b) yield potentials of the tested feedstock substrates calculated according to Weißbach (2009). Calculations are based on ash content, crude fiber content, and enzyme solubility of harvested substrates before ensiling. Numbers in the bar indicate the standing age of the biomass stock. Substrates nomenclature: FM100 = pure biomass from flowering stripes, FM67 = mixture of 67% flower stripe’s biomass and 33% silage maize, FM33 = 33% flower stripe’s biomass and 67% silage maize, ZM100 = pure silage maize, YSC99 = 99% yellow sweet clover. [file Data_Sheet_2.PDF]
